# Voluntary Medical Male Circumcision: A Cross-Sectional Study Comparing Circumcision Self-Report and Physical Examination Findings in Lesotho

**DOI:** 10.1371/journal.pone.0027561

**Published:** 2011-11-29

**Authors:** Anne Goldzier Thomas, Bonnie Robin Tran, Marcus Cranston, Malerato Cecilia Brown, Rajiv Kumar, Matsotetsi Tlelai

**Affiliations:** 1 Department of Defense HIV/AIDS Prevention Program, Naval Health Research Center, San Diego, California, United States of America; 2 United States Air Force, Department of Defense, Keesler Air Force Base, Mississippi, United States of America; 3 United States President's Emergency Plan for AIDS Relief—Lesotho, United States Embassy, Maseru, Lesotho; 4 Indian Armed Forces, Pune, India; 5 Lesotho Defence Force, Maseru, Lesotho; Centers for Disease Control and Prevention, United States of America

## Abstract

**Background:**

Overwhelming evidence, including three clinical trials, shows that male circumcision (MC) reduces the risk of HIV infection among men. However, data from recent Lesotho Demographic and Health Surveys do not demonstrate MC to be protective against HIV. These contradictory findings could partially be due to inaccurate self-reported MC status used to estimate MC prevalence. This study describes MC characteristics among men applying for Lesotho Defence Force recruitment and seeks to assess MC self-reported accuracy through comparison with physical-examination-based data.

**Methods and Findings:**

During Lesotho Defence Force applicant screening in 2009, 241 (77%) of 312 men, aged 18–25 y, consented to a self-administered demographic and MC characteristic survey and physician-performed genital examination. The extent of foreskin removal was graded on a scale of 1 (no evidence of MC) to 4 (complete MC). MC was self-reported by 27% (*n* = 64/239) of participants. Of the 64 men self-reporting being circumcised, physical exam showed that 23% had no evidence of circumcision, 27% had partial circumcision, and 50% had complete circumcision. Of the MCs reportedly performed by a medical provider, 3% were Grade 1 and 73% were Grade 4. Of the MCs reportedly performed by traditional circumcisers, 41% were Grade 1, while 28% were Grade 4. Among participants self-reporting being circumcised, the odds of MC status misclassification were seven times higher among those reportedly circumcised by initiation school personnel (odds ratio = 7.22; 95% CI = 2.29–22.75).

**Conclusions:**

Approximately 27% of participants self-reported being circumcised. However, only 50% of these men had complete MC as determined by a physical exam. Given this low MC self-report accuracy, countries scaling up voluntary medical MC (VMMC) should obtain physical-exam-based MC data to guide service delivery and cost estimates. HIV prevention messages promoting VMMC should provide comprehensive education regarding the definition of VMMC.

## Introduction

With an estimated 2.6 million incident HIV infections in 2009 and 33.3 million prevalent infections worldwide, the need for effective HIV prevention has never been more critical [1]. In observational studies [2]–[4] and randomized clinical trials [5]–[7], male circumcision (MC) has been shown to decrease HIV acquisition in men. Responding to these findings, the World Health Organization (WHO) published recommendations supporting voluntary medical male circumcision (VMMC) for HIV prevention in countries with high HIV and low MC prevalence [8].

Many countries are planning or already delivering VMMC as a component of comprehensive HIV prevention services. However, some countries may be hesitant to scale up VMMC because of nationally representative survey results demonstrating higher HIV prevalence among those who report being circumcised [9]–[11]. MC impact modeling shows that reaching 80% MC coverage in a 5-y time frame will have the most substantial impact on HIV incidence. These models rely on nationally representative MC prevalence data [12]. However, MC prevalence estimates available to planners and policy makers, such as those in the Demographic Health Survey or other nationally representative cross-sectional surveys, are based on self-reported MC status. Several studies comparing MC status classification in diverse settings, as determined by self-reporting and physical examination, have shown large reporting discrepancies between these different data collection modalities [13]–[15].

The Joint United Nations Programme on HIV/AIDS estimates that 24.5% of those aged 15–49 y living in the Kingdom of Lesotho—which has a population of 1.9 million—are infected with HIV [1]. The 2004 Lesotho Demographic and Health Survey (LDHS) found that 48% of men aged 15–59 y self-identified as being circumcised. Contrary to most studies correlating lower HIV prevalence with higher prevalence of MC [16], the 2004 LDHS reported 23% HIV prevalence among men reporting MC and 15% for those reporting no MC [9]. Similarly, the 2009 LDHS reported 21% HIV prevalence among men reporting MC and 16% among those reporting no MC [10]. These data, showing higher HIV prevalence in those self-reporting MC, are similar to findings from other countries, including Malawi [11]. These study results could be explained by misclassification of MC status due to self-report, or lack of adjustment for confounders such as MC occurring after sexual debut [17]–[19], traditional MC practices that promote the spread of HIV, such as the reuse of unsterilized MC cutting instruments on multiple males [20], [21], or males having sex just after being circumcised, before the wound has completely healed [17]. Thus, further studies exploring the concordance of self-reported circumcision status with physical examination are needed to better understand the factors associated with discrepant reporting and the magnitude of inaccuracy.

This paper presents findings from a cross-sectional study estimating the prevalence of MC and exploring factors associated with discrepant reports of MC status in a sample of young adult men undergoing physical examination during the Lesotho Defence Force (LDF) recruitment process.

## Methods

From March to April 2009, all men undergoing physical examination for voluntary recruitment into the LDF were invited to participate in the study. The applicants were aged 18 y or older and came from all ten districts of Lesotho.

Human subjects participated in this study after giving their free and informed consent. This research has been conducted in compliance with all applicable Federal Regulations governing the Protection of Human Subjects in Research. Institutional review boards in the United States (Naval Health Research Center, San Diego, California) and Lesotho (Ministry of Health, Lesotho) approved the study prior to data collection. Potential participants received written and verbal information about the purpose and methods of the study and were given an opportunity to ask any questions about their possible participation. All men undergoing entry physicals (*n* = 312) were invited to participate in the study, and 241 (77.2%) agreed to participate and provided written informed consent.

LDF nurses and doctors were trained on the study protocol using standardized materials, including how to correctly classify the different grades of MC using the grading scale [22] (Table 1) and a graphic assessment tool. LDF nurses briefed the applicants about the study and conducted the informed consent process. Study participants were not provided any HIV prevention education or information regarding MC prior to completing a brief self-administered survey about their age, birthplace, marital status, education level, and religious affiliation. In addition, participants were asked about their circumcision status with the question “Are you circumcised?” The response choices were “yes” or “no.” If an individual answered “yes,” he was prompted on the survey to provide age at circumcision, town/area of circumcision, district of circumcision, attendance at an initiation school (where a traditional circumciser would provide MC), the circumcision setting (hospital/medical clinic, church or place of worship, initiation school, home, or other), and the circumcision provider (doctor or medical personnel, religious leader, initiation school personnel, family member/relative, or other).

**Table 1:** Grading criteria for extent of male circumcision.

Grade	Description
1	Foreskin covers one-half or more of the glans; completely uncircumcised
2	Foreskin is past the sulcus, but covers less than one-half of the glans
3	Foreskin is not past the sulcus, but can be extended past the sulcus to cover one-half of the glans without compressing the glans
4	Foreskin is completely absent; completely circumcised

During the physical examination, with a study nurse present, a medical doctor examined the penis for extent of circumcision, using a four-point scale to classify the foreskin from completely uncircumcised (Grade 1) to completely circumcised (Grade 4) (Table 1). After the physical examination, the physician placed study materials into a sealed envelope, which was then collected by the study coordinator. All study materials were stored securely and made available only to study personnel.

Of the 241 consented participants, data on two were missing the variable of interest, self-reported MC status, and were excluded from the analysis. Descriptive statistics were computed for the remaining 239 men. Frequency counts and percentages were tabulated for categorical variables, and means and standard deviations were calculated for continuous variables. To assess population sample representativeness, district comparisons of the participant population were made with the 2006 Lesotho census and the 2004 LDHS, which used the 1996 Lesotho census for the population sampling frame.

Pearson's chi-square tests (for categorical variables) were used to determine any significant difference (*p*<0.05) in grade of MC by circumcision characteristics. Fisher's exact tests were used when expected cell frequencies were less than five. A one-way analysis of variance was used to determine significant differences in means between two or more groups.

Prevalence of MC by self-report was calculated by dividing the number of men who answered “yes” to the question whether they were circumcised by the total number of men who answered the question. Prevalence of complete MC as determined by physical examination was calculated by dividing the number of men who were classified by the physician as having a Grade 4 circumcision by the total number of men who underwent a physical examination.

Simple logistic regression analysis was used to examine the associations of demographic and MC characteristics with inaccurate reporting of MC status among participants who self-reported being circumcised. Participants' responses were classified as “inaccurate” if they reported they were circumcised (answered “yes” to the question “Are you circumcised?”) and were subsequently classified as Grade 1, 2, or 3 by a physician. Participants' responses were classified as “accurate” if they reported they were circumcised and were subsequently classified as Grade 4 by a physician. A sub-analysis was also conducted in which participants with MC Grades 2 and 3 were excluded, as some may argue that those with Grades 2 and 3 are circumcised, as they do have evidence of some level of circumcision.

All statistical tests were two-tailed and were performed using SAS statistical software, version 9.2 (SAS Institute).

## Results

Demographic characteristics of study participants (*n* = 239) are presented in Table 2. The mean age was 21.5 y, with a range of 18–25 y. Most (88.2%) of the men were single; 9.6% reported being married, and 2.2% were cohabiting. The majority (86.5%) of study participants had completed secondary/high school, as compared with 28.0% of the 2004 LDHS male population. Religious affiliation was mixed and similar to the 2004 LDHS sample. Participants were from all ten districts in the country, with a distribution representative of the 2006 Lesotho census and the 2004 LDHS (see Table S1).

**Table 2:** Demographic characteristics of study participants (*n* = 239).

Demographic Characteristic	Value
**Age in years**	21.5±1.4
**Current marital status**	
Married	22 (9.6)
Living together	5 (2.2)
Not in union	202 (88.2)
**Education**	
Vocational/tech primary	4 (1.7)
Secondary/high	205 (86.5)
Vocational/tech secondary	14 (5.9)
College	14 (5.9)
**Religion**	
Roman Catholic	115 (48.3)
Lesotho Evangelical	66 (27.7)
Anglican	23 (9.7)
Other Christian	29 (12.2)
None/other	5 (2.1)

All values are *n* (percent), except for age, which is given as mean ± standard deviation.

Of 239 participants, 175 (73.2%) reported that they were not circumcised (Table 3). Of these men, physical examination revealed that 95.4% showed no evidence of circumcision, and 4.0% were partially circumcised (Grades 2 and 3) or had naturally shorter foreskins. Of this group that reported not being circumcised, one participant (0.6%) was classified as having a complete circumcision (Grade 4).

**Table 3:** Self-reported male circumcision characteristics by grade of circumcision as determined by physical examination.

MC Characteristic	Total, *n* (%)^a^	Extent of Circumcision by Physical Exam, *n* (%)^b^				*p*-Value
		Grade 1	Grade 2	Grade 3	Grade 4	
**Self-reported circumcision status (** ***n*** ** = 239)**						<0.001^c^
Circumcised	64 (26.8)	15 (23.4)	11 (17.2)	6 (9.4)	32 (50.0)	
Not circumcised	175 (73.2)	167 (95.4)	5 (2.9)	2 (1.1)	1 (0.6)	
**Age at MC in years, mean ± SD (** ***n*** ** = 60)** d	17.1 (4.5)	18.4 (2.6)	16.8 (3.5)	15 (5.2)	16.9 (5.3)	0.50^e^
**Provider of MC (** ***n*** ** = 61)** d						<0.001^c^
Doctor/medical personnel	30 (49.2)	1 (3.3)	4 (13.3)	3 (10.0)	22 (73.3)	
Initiation school personnel	29 (47.5)	12 (41.4)	7 (24.1)	2 (6.9)	8 (27.6)	
Religious leader	1 (1.6)	0 (0)	0 (0)	0 (0)	1 (100)	
Other	1 (1.6)	1 (100)	0 (0)	0 (0)	0 (0)	
**Setting of MC (** ***n*** ** = 62)** d						<0.001^c^
Hospital/medical clinic	30 (48.4)	1 (3.3)	4 (13.3)	4 (13.3)	21 (70.0)	
Initiation school	31 (50.0)	14 (45.2)	6 (19.4)	2 (6.5)	9 (29.0)	
Home	1 (1.6)	0 (0)	0 (0)	0 (0)	1 (100)	

Due to rounding, percentages may not add up to 100.

a Column percentages are presented.

b Row percentages are presented.

c Results of chi-square analysis.

d Missing participant responses.

e Results of one-way analysis of variance.

SD, standard deviation.

Sixty-four participants (26.8%) reported that they were circumcised (Table 3). The mean reported age at the time of circumcision was 17.1 y, which did not differ significantly by grade of MC (*p* = 0.50). In addition, there was no significant difference in mean age at MC among those circumcised in a traditional (15.9 y) versus medical (17.7 y) setting (*p* = 0.14; data not shown). Among those who reported being circumcised, nearly half of the circumcisions were performed by a medical professional (*n* = 30/61; 49.2%) and half (*n* = 29/61; 47.5%) by initiation school personnel. Similarly, approximately half (*n* = 30/62; 48.4%) of the self-reported MCs were performed in a medical setting, and half (*n* = 31/62; 50.0%) were performed in initiation school settings.

Of those who reported being circumcised (*n* = 64), physical examination revealed that 23.4% had no evidence of MC (Grade 1), 26.6% had evidence of partial MC (Grades 2 and 3), and 50.0% were classified as having complete foreskin removal (Grade 4). The grade of MC differed significantly by MC provider (*p*<0.001). Among the MCs reported to be performed by medical personnel, 3.3% were classified as Grade 1, while 13.3% were classified as Grade 2, 10.0% were classified as Grade 3, and 73.3% were classified as Grade 4. The MCs reported to be performed by initiation school personnel were classified as follows: Grade 1 (41.4%), Grade 2 (24.1%), Grade 3 (6.9%), and Grade 4 (27.6%). The association of grade of MC and the setting of the MC was also found to be statistically significant (*p*<0.001), with MCs performed at an initiation school more likely to be reported as Grade 1 or 2 than those performed in a medical setting (Table 3). The three districts with the highest percentages of discrepant self-reported MC status were Butha-Buthe (21.4%), Qacha's Nek (14.3%), and Quthing (14.3%) (see Table S2).

Overall, the prevalence of MC as determined by self-report was 26.8% (*n* = 64/239). The prevalence of Grade 4 MC (complete MC) as determined by physical examination was 13.8% (*n* = 33/239) (Table 3). The difference between the self-reported MC prevalence and the physician-classified prevalence of Grade 4 MC is 13.0%.

Results of simple logistic regression analysis examining factors significantly associated with inaccurate MC reporting among participants who self-reported MC are presented in Table 4. Age, current marital status, education, and age at MC were not significantly associated with inaccurate reporting of MC (*p*>0.05). MC provider and setting were significantly associated with inaccurate reporting of MC. The odds of inaccurate reporting of MC were seven times higher among participants who reported that their MC was performed by initiation school personnel than among those who reported that their MC was performed by a doctor or medical personnel (odds ratio [OR]  = 7.22; 95% CI = 2.29–22.75). Similarly, the odds of inaccurate reporting of MC was almost six times higher among participants who reported that their MC was conducted at an initiation school than among those who reported that their MC was conducted at a hospital or medical clinic (OR = 5.70; 95% CI = 1.90–17.14). In the sub-analysis excluding participants with MC Grades 2 and 3, significant, elevated odds of inaccurate MC reporting were also observed among those who reported their MC was performed by initiation school personnel (OR = 33.0; 95% CI = 3.68–296.23) or conducted at an initiation school (OR = 32.7; 95% CI = 3.72–287.21) (data not shown).

**Table 4:** Univariate associations of demographic and male circumcision characteristics with inaccurate self-reporting of MC among participants who self-reported “yes” to MC (*n* = 64).

Characteristic	Inaccurate Self-Reporting of MC		
	OR	(95% CI)	*p*-Value
**Age in years**	0.88	(0.60–1.28)	0.51
**Current marital status**			
Not in union	1.0		0.78
Married	0.58	(0.13–2.67)	
Living together	0.96	(0.06–16.21)	
**Education**			
Secondary/high	1.0		0.70
Vocational/tech secondary	0.47	(0.08–2.75)	
College	0.93	(0.06–15.6)	
**Age at MC in years**	1.02	(0.91–1.14)	0.77
**Provider of MC** a			
Doctor/medical personnel	1.0		0.001
Initiation school personnel	7.22	(2.29–22.75)	
**Setting of MC** b			
Hospital/medical clinic	1.0		0.002
Initiation school	5.70	(1.90–17.14)	

Results of simple logistic regression analysis.

a Excludes participants who reported religious leader (*n* = 1) or other (*n* = 1) as provider of MC.

b Excludes participants who reported home as setting of MC (*n* = 1).

## Discussion

The results from this cross-sectional study of MC among male LDF applicants provide further evidence that categorizing MC status by self-report is highly prone to error. Upon physical examination, only 50% of participants self-reporting circumcision showed evidence of complete circumcision, and another 27% showed evidence of only partial circumcision. The prevalence of self-reported MC (27%) in this study is also much lower than the prevalence found in the 2004 and 2009 LDHS studies (48% and 52%, respectively). The discrepancies in self-reported MC status found in this study suggest that data from population-based surveys evaluating associations between MC and risk of HIV infection should be interpreted with caution. The magnitude of inaccurate self-reported MC status could be sufficient to explain the apparent correlation of higher rates of HIV with higher rates of MC, as seen in the Lesotho and Malawi Demographic Health Surveys. However, other factors could also explain this observation, including lack of adjustment for confounders, such as MC occurring after sexual debut and traditional MC practices that may promote the transmission of HIV.

This study has some limitations. The LDF recruit applicant population was similar to the 2004 LDHS population in terms of district of birth and religion. Thus, for MC practices and MC-related sexual practices related to local cultural or religious affiliation, the LDF applicant study population is representative of the national population. But the LDF applicants were more educated and of a more restricted age range than the general population. However, this does not appear to have biased the study results, as the average MC prevalence in the LDHS is similar across all men aged 20 y and older (57.9%). However, higher education may be associated with increased medical MC as compared with traditional MC, because of an increased appreciation for the medical risks of MC performed in traditional settings. Alternatively, increased educational attainment might be a marker of increased family income to pay for a medical circumcision. It is unclear whether medical MC is ultimately more expensive than traditional MC, since traditional MC may include costs for hosting community celebrations as well as payment for the circumciser and initiation school [17]. Another limitation to this study is that the physicians were not blinded to the participants' reported MC status, nor were the physicians' assessments of the MC grades validated, which may have resulted in misclassification. Although we cannot exclude the possibility of MC grade misclassification, the grading scheme was relatively simple and the rates of misclassification were most likely low.

Despite these limitations, this study has many strengths. Among them is the short time frame that was required for the study to be implemented; the data was collected in approximately four weeks during the LDF applicant examinations. This short time frame is especially important as educational campaigns and news of MC as an HIV prevention modality become more common. Similarly, study personnel may have implemented the protocol with higher conformity during this short time frame. Offering study participation to men already undergoing physical examination including genital exam may have reduced non-respondent bias. The regional and religious representativeness of the study population are other key strengths, as these demographic characteristics are often related to MC practices. The higher educational status of the LDF applicants may also be a strength, as these individuals may be more likely to provide an accurate report of their circumcision status.

Discrepancies in self-reported MC status have important implications for planning VMMC scale-up, communication, and education for HIV prevention, as reliance on self-reported MC status may underestimate the volume of surgical intervention required and not accurately identify those individuals for which the intervention is indicated. While 26.8% of the participants self-reported being circumcised, the prevalence of complete MC as estimated by physical examination was only 13.8%. This means that if self-reported data were used to estimate the need for MC in this population, the need would be underestimated by 13.0%. If these findings are applied to Lesotho as a whole, the difference between the 52% self-reported MC prevalence found in the 2009 LDHS and the 14% MC prevalence found in this study population increases the national need for VMMC by 38%. The prevention effect of VMMC in reducing HIV incidence in Lesotho may also be vastly underestimated. Furthermore, these data show that while most of the discrepant results were among those who reported undergoing traditional MC, a substantial percentage of the physician-performed VMMCs were incomplete (*n* = 8/30; 26.6%), which suggests that additional surgical training may be necessary to ensure adequate VMMC results to improve HIV prevention outcomes [23].

This study adds to the evidence that determining MC status through self-report is prone to result in misclassification [24], [25]. Even among participants who reported not being circumcised, about 5% were partially or completely circumcised. The reasons for inaccurate self-report may be that (1) survey tools and methods do not currently allow for more than dichotomous (yes/no) categorization and do not capture all aspects of MC (such as the level of foreskin removal or nuances of traditional circumcision), (2) there is a misunderstanding of the meaning of medical circumcision as compared with traditional circumcision, or (3) there is a desire to maintain secrecy about initiation rites. Future studies seeking to improve MC self-report may benefit from the addition of partial MC categories, along with graphics depicting all four grades of male circumcision.

The associations of demographic and MC characteristics with inaccurate reporting of MC status in this study were also examined. The only factors shown to be significantly associated with inaccurate reporting were having MC performed by initiation school personnel or conducted at an initiation school. This association was significant even when those with Grade 2 and 3 MCs were removed from the analysis. In many countries, including Lesotho, South Africa, Malawi, Namibia, Kenya, and Uganda, non-medical MC is commonly performed during attendance at a traditional initiation school [26], and undergoing MC in this setting is a rite of passage to manhood. Underscoring this point, the word in Sesotho for going through the initiation process, “lebollo,” is very similar to the word for circumcision. Thus, a male who has attended an initiation school may report that he has been circumcised even if the foreskin was only cut, or only part of the foreskin was removed. These results provide compelling evidence that specific VMMC communication campaigns must include factual information describing or graphically representing the penile foreskin, so that VMMC is understood to mean the complete removal of the foreskin and is not conflated with traditional MC practices, such as those conducted at initiation schools.

As nations with high HIV prevalence begin to act on WHO recommendations for VMMC programs, the need for accurate MC prevalence data becomes even more critical. Thus, until further research can document improved methods for obtaining accurate self-reported MC data, all assessments of MC and HIV prevalence, as well as projections for VMMC interventions, should be informed by physical-exam-based data.

## Supporting Information

Table S1 **Study population: district of residence (** ***n*** **= 239).**
(DOCX)

Table S2 **District of male circumcision by grade of circumcision as determined by physical examination (** ***n*** **= 64).**
(DOCX)

## Abbreviations

LDF: Lesotho Defence Force
LDHS: Lesotho Demographic and Health Survey
MC: male circumcision
OR: odds ratio
VMMC: voluntary medical male circumcision
WHO: World Health Organization
