# Supplementary material for: Voluntary Medical Male Circumcision: A Cross-Sectional Study Comparing Circumcision Self-Report and Physical Examination Findings in Lesotho
Source: PLoS One. 2011 Nov 29;6(11):e27561. doi: 10.1371/journal.pone.0027561 (PMC3226626; doi:10.1371/journal.pone.0027561)
Supplement: Table S1 — Study population: district of residence ( n = 239). (DOCX) [file pone.0027561.s001.docx]

**Table S1.** Study population: district of residence (*n* = 239).

| **District of Residence** | **Value** |
| --- | --- |
| Berea | 33 (13.9) |
| Butha-Buthe | 18 (7.6) |
| Leribe | 31 (13.0) |
| Mafeteng | 21 (8.8) |
| Maseru | 55 (23.1) |
| Mohale’s Hoek | 18 (7.6) |
| Mokhotlong | 12 (5.0) |
| Qacha’s Nek | 13 (5.5) |
| Quthing | 16 (6.7) |
| Thaba-Tseka | 21 (8.8) |

All values are *n* (percent).
