# Supplementary material for: Voluntary Medical Male Circumcision: A Cross-Sectional Study Comparing Circumcision Self-Report and Physical Examination Findings in Lesotho
Source: PLoS One. 2011 Nov 29;6(11):e27561. doi: 10.1371/journal.pone.0027561 (PMC3226626; doi:10.1371/journal.pone.0027561)
Supplement: Table S2 — District of male circumcision by grade of circumcision as determined by physical examination ( n = 64). (DOCX) [file pone.0027561.s002.docx]

**Table S2. District of male circumcision by grade of circumcision as determined by physical examination (n=64)***

|  |  | **Extent of Circumcision by Physical Exam** | | | |  |
| --- | --- | --- | --- | --- | --- | --- |
|  | **Total** | **Grade 1** | **Grade 2** | **Grade 3** | **Grade 4** |  |
|  | **n (%)**^†^ | **n (%)**^‡^ | **n (%)**^‡^ | **n (%)**^‡^ | **n (%)**^‡^ | **p-value** |
| **District of MC** (n = 58)^§^ |  |  |  |  |  | 0.19^¶^ |
| Berea | 4 (6.9) | 1 (25.0) | 1 (25.0) | 0 (0) | 2 (50.0) |  |
| Butha-Buthe | 12 (20.7) | 3 (25.0) | 1 (8.3) | 0 (0) | 8 (66.7) |  |
| Leribe | 4 (6.9) | 1 (25.0) | 1 (25.0) | 0 (0) | 2 (50.0) |  |
| Mafeteng | 4 (6.9) | 1 (25.0) | 0 (0) | 0 (0) | 3 (75.0) |  |
| Maseru | 10 (17.2) | 0 (0) | 1 (10.0) | 4 (40.0) | 5 (50.0) |  |
| Mohale’s Hoek | 3 (5.2) | 1 (33.3) | 0 (0) | 1 (33.3) | 1 (33.3) |  |
| Mokhotlong | 5 (8.6) | 1 (20.0) | 2 (40.0) | 0 (0) | 2 (40.0) |  |
| Qacha’s Nek | 5 (8.6) | 2 (40.0) | 0 (0) | 0 (0) | 3 (60.0) |  |
| Quthing | 6 (10.3) | 2 (33.3) | 3 (50.0) | 1 (16.7) | 1 (0) |  |
| Thaba-Tseka | 4 (6.9) | 1 (25.0) | 1 (25.0) | 0 (0) | 2 (50.0) |  |
| Kwa Zula Natal (South Africa) | 1 (1.7) | 1 (100) | 0 (0) | 0 (0) | 0 (0) |  |

* Due to rounding, percentages may not add up to 100.

† Column percents are presented.

‡ Row percents are presented.

§ Missing participant responses.

¶ Results of chi-square analysis.
